# Novel insights on premature progesterone elevation: a mini-review

**DOI:** 10.5935/1518-0557.20210096

**Published:** 2022

**Authors:** Alfredo Cortés-Vazquez, Cristabel Escobosa, Alfredo L. Cortés-Algara, Jesús D. Moreno-García

**Affiliations:** 1 Centro Médico Nacional 20 de Noviembre, Mexico City, Mexico

**Keywords:** progesterone, rise, IVF, premature, luteinization, embryo

## Abstract

*In vitro* fertilization (IVF) success involves ovarian stimulation with conventional or mild stimulation protocols aimed to yield an adequate number of mature oocytes. These oocytes can be further fertilized and generate good quality embryos to be transferred, ideally in the same cycle. Since 2013, following the results of a meta-analysis including more than 60,000 IVF cycles, the negative effects of premature progesterone elevation on reproductive outcomes have been demonstrated. Nowadays, evidence is emerging on the limited regulation on serum progesterone values, demonstrating significantly lower live birth rates in patients with either low (<0.5ng/ml) late follicular phase progesterone or higher levels decreasing sharply. This review discusses and summarizes the different mechanisms of progesterone elevation and its impact on IVF treatments. Different approaches to diminish the impact of progesterone on fertility outcomes are also addressed.

## INTRODUCTION

*In vitro* fertilization (IVF) success involves ovarian stimulation with conventional or mild stimulation protocols aimed to yield an adequate number of mature oocytes. These oocytes can be further fertilized and generate good quality embryos to be transferred, ideally in the same cycle. During controlled ovarian stimulation, oocyte maturation occurs *in vivo* after ovulation trigger, with either human chorionic gonadotropin (hCG) or a gonadotropin-releasing hormone (GnRH) agonist, mimicking the luteinizing hormone surge that occurs during the menstrual cycle (Griffin *et al*., 2014). Progesterone plays a critical role in IVF success, since it is a critical regulator of endometrial receptivity. Since 2013, following the results of a meta-analysis including more than 60,000 IVF cycles, the negative effects of premature progesterone elevation on reproductive outcomes were demonstrated (Venetis *et al*., 2013). Evidence has since emerged to show the limited regulation on serum progesterone values and reveal the significantly lower live birth rates in patients with either low (<0.5ng/ml) late follicular phase progesterone or higher levels decreasing sharply (Arvis *et al*., 2019). In the pre-gonadotropin-releasing hormone analogue era, late follicular phase elevations of serum progesterone resulted from a premature luteinizing hormone (LH) elevation during controlled ovarian stimulation, correctly defined as *premature luteinization* (Al-Azemi *et al*., 2012). GnRH analogues made LH suppression feasible and diminished cycle cancellations due to premature luteinization. Unfortunately, elevated progesterone levels on the day of ovulation trigger still occur in 5% to 50% of IVF cycles, despite optimal LH suppression (Huang *et al*., 2016). Nowadays, this process is defined as a rise in serum progesterone concentration towards the end of the follicular phase above a threshold concentration, usually set arbitrarily (Van Vaerenbergh *et al*., 2011).

The exact mechanism by which progesterone rises on the late follicular phase is currently a matter of debate. Moreover, it is still difficult to define the optimal cut-off level in stimulated IVF cycles, with thresholds varying from 0.8 to 2.0ng/ml (Drakopoulos *et al*., 2019). The most commonly used threshold is 1.5ng/ml on ovulation trigger day, supported by the presence of a significant change in gene profile expression in endometrial tissue among infertile patients (Labarta *et al*., 2011). However, caution is strongly recommended in setting the progesterone cut-off level. There is high variability between assays used for progesterone measurement. It is essential to notice that progesterone-derived metabolites (e.g. five alpha-pregnanedione and 20 alpha-dihydroprogesterone) are present in significant amounts in circulation. These metabolites are chemically very similar to progesterone and might lead to a cross-reaction with the progesterone anti-serum on direct immunoassays (de Ziegler *et al*., 2018).

In this review, different mechanisms of progesterone elevation and its impact on IVF treatments are discussed and summarized. Different approaches to diminish the impact of progesterone on fertility outcomes are also addressed.

## MECHANISMS OF PROGESTERONE ELEVATION DURING OVARIAN STIMULATION

During conventional and mild ovarian stimulation protocols, estrogen and progesterone levels reach supraphysiological levels due to multiple follicular growth derived from continuous stimulation required to maintain high FSH serum concentration above the threshold needed for follicle development. In a natural menstrual cycle, estrogens and progesterone are secreted and reach relatively low serum levels before ovulation, as shown in Table 1. In response, estradiol mainly regulates the isoform mixture of secreted FSH and down-regulates pituitary expression of the glycosyltransferases responsible for the less-acidic FSH isoforms as ovulation approaches, consequently affecting FSH half-life (Yding Andersen, 2002). Once estrogen exceeds a threshold, it produces a positive feedback on LH surge, which takes on average 36 hours to complete (de Ziegler *et al*., 2007). Interestingly, LH stimulates theca cell receptors by inducing the production of Cytochrome P450 CYP 17 enzymatic complex (17 hydroxylase and 17-20 lyase activities) responsible for the conversion of progesterone (via delta four pathway) and pregnenolone (delta five pathway) into 17 hydroxylated products and androgens (Hugues, 2012). By mid-cycle and alongside LH surge, meiotic inhibition is invalidated. The oocyte can undergo germinal vesicle (GV) breakdown to produce a haploid metaphase II oocyte, with a mature cytoplasm that can be fertilized and develop into an embryo (Dumesic *et al*., 2015).

**Table 1.** Plasma concentrations, ovarian secretion rates, and total production rates of estradiol, E1, and progesterone during the various phases of the menstrual cycle.

Steroid	Early follicular			Late follicular		
	Plasma concentration (pg/mL)	Secretion rate (mg/day)	Production rate (mg/day)	Plasma concentration (pg/mL)	Secretion rate (mg/day)	Production rate (mg/day)
**Estradiol**	60	0.07	0.08	330-700	0.4-0.8	0.44-0.94
**E1**	50	0.08	0.11	150-300	0.25-0.5	0.33-0.66
**Progesterone**				0.39-1.30		

Modified from Stanczyk et al. (2013)

In IVF treatments, the criteria for the initial FSH dose is still unclear, and clinicians often choose the exogenous FSH starting dose according to the patient's clinical history and characteristics, primarily taking into account the outcome of prior stimulations and, secondly, the age of the female patient and her ovarian reserve markers. From 36.8% to 52.4% of the patients have received an excessive FSH starting dose (Papaleo *et al*., 2016). This excessive FSH dose can provide different outcomes according to age and ovarian response. In a first scenario, on a patient with good ovarian reserve and appropriate response, excessive FSH might lead to multifollicular development, resulting in a large number of follicles, with each follicle contributing to progesterone elevation in systemic circulation (Lawrenz & Fatemi, 2017). Also, it is vital to keep in mind that FSH has a direct stimulation effect on granulosa cell lines and triggers the expression of 3-beta-hydroxysteroid dehydrogenase (3-beta-HSD) and progesterone biosynthesis in these cells, which then increases the conversion of pregnenolone into progesterone in a dose-dependent fashion. Likewise, FSH cannot stimulate 17-alpha-hydroxylase in granulosa cell line.

Along with continuously high FSH levels in the absence of LH support may exceed the ovarian ability to transform them into estrogen pathway efficiently (Lawrenz *et al*., 2018a). In a regular menstrual cycle, intrafollicular progesterone concentrations go up as the follicular diameter increases and get as far as the equivalent to 1,000 times the circulating levels in preovulatory follicles (de Ziegler *et al*., 2018). The previously mentioned mechanism delays the conversion of progesterone into androgens in theca cells, promoting their accumulation and leak into the systemic circulation (Lawrenz *et al*., 2018a). These findings sustain the theory that intense and constant ovarian stimulation close to the late follicular phase may be the primary cause of premature progesterone elevation (Drakopoulos *et al*., 2019).

In a second scenario, women with poor ovarian response (POR) tend to receive high FSH doses, although they respond with a limited number of follicles, which does not correlate with ovarian steroidogenic activity. As a matter of fact, low gonadotropin responses are often distinguished by serum estradiol levels achieved through a 7 to 14 day controlled ovarian stimulation period (Luborsky *et al*., 2002). Ageing oocytes display several impaired quality markers, such as a compromised balance between mitochondria-smooth endoplasmic reticulum (MSER) aggregates and mitochondria-vesicle complexes, along with other ultrastructural markers such as specific defects in spindle location, chromosome alignment, and tubulin distribution in aged human oocytes (Bianchi *et al*., 2015). There is some evidence suggesting how paracrine and autocrine mechanisms in the oocyte-cumulus complex might protect the preovulatory follicle, which comprises progesterone-elevation inhibition and follicular luteinization. In the presence of an ageing oocyte and a low ovarian reserve, these checkpoints are deficient (Younis, 2019). These conclusions are supported by the fact that messenger RNA (mRNA) expression of steroidogenic enzymes (aromatase and 17-beta-hydroxysteroid dehydrogenase) is significantly lower in women of advanced age (Wu *et al*., 2015).

**Figure 1:**
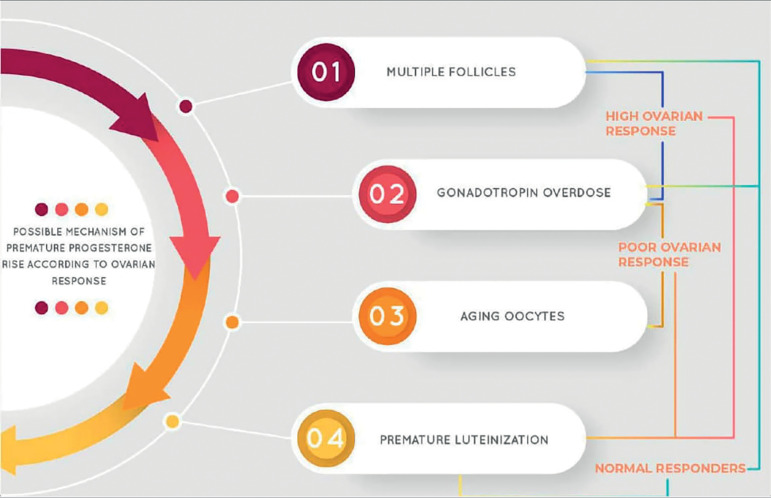
Possible mechanism of premature progesterone rise according to ovarian response.

Besides ovarian steroid secretion, some authors noted that progesterone and androgens (testosterone and androstenedione) peak in the early morning during ovarian stimulation for IVF. These sex hormone elevations occur in line with circadian adrenal function. Figure 1 summarizes the main mechanisms that possibly act on premature progesterone rise.

## STIMULATION LENGTH AND INTENSITY

Some authors claimed that LH deficiency might cause premature progesterone elevation, and that LH support might reduce the risk of it happening. The idea was dismissed based on the fact that LH activity increases progesterone production during the follicular phase. (Lawrenz *et al*., 2018b). This point of view supports the findings of a systematic review published in 2012, which demonstrated that FSH alone or in combination with recombinant LH (rLH) did not produce consistent serum progesterone increases. Additionally, the FSH-hCG combination may be linked to ascending serum progesterone levels (Hugues, 2012). Today, evidence from systematic reviews supports the indication of LH supplementation to patients with adequate ovarian reserve parameters, individuals with unexpected poor response to ovarian stimulation with rFSH monotherapy, and patients aged 36-39 years (Alviggi *et al*., 2018).

Recent evidence from post hoc analysis of the data from the ENGAGE and PURSUE trials indicates that corifollitropin alfa was linked to significantly lower incidence of progesterone elevation than recombinant FSH (rFSH). The pharmacokinetic profile of corifollitropin shows higher FSH activity during the first two days, followed by decreased FSH activity. This pharmacokinetic pattern mimicked a step-down protocol and showed a marked decrease (5.4% *vs*. 18.3%) in premature progesterone incidence (Lawrenz *et al*., 2016). Also, corifollitropin alfa mimics the natural cycle regulation on FSH half-life by modifying the molecule's acidity, as discussed previously. In our experience, high FSH doses do not correlate well with the number of retrieved oocytes in conventional ovarian stimulation protocols (Vázquez *et al*., 2021). It is worth noting that excessive gonadotropin stimulation might impair the oocyte's developmental competence due to alterations in spindle formation, which create a path for aneuploidy (Bianchi *et al*., 2015). Therefore, we encourage the use of nomograms and predictive models to help clinicians guide their clinical decisions. In a mathematical model, Nisal *et al*. (2020) demonstrated that predictive models can achieve a significantly lower dosage for each patient and that almost 98% of the patients had higher mature follicles for the optimal control profile than the physician-specified dosage. Several authors have found similar evidence and proposed the use of a cost-effective way to customize assisted reproduction technology treatments (Chalumeau *et al*., 2018; van Tilborg *et al*., 2017; Allegra *et al*., 2017).

Regarding the length of ovarian stimulation, it should be noted that serum progesterone levels rise linearly with both follicle size and maturity (Schneyer *et al*., 2000). Therefore, delaying the ovulation trigger might lead to further follicle growth and higher serum progesterone levels. Harada *et al*. (1996) described in a retrospective study that altering the timing of ovulation trigger when an insidious rise in progesterone is detected may lead to improved embryo quality and implantation rates. A *post hoc analysis* of the Ensure study demonstrated that prolonged and intense stimulation were associated with a higher incidence of premature progesterone rise on the trigger day (Lawrenz *et al*., 2018c). Decreased immature oocyte rate is also a concern in IVF cycles, with some cycles reaching a 30% immaturity rate. Evidence also shows that insufficient gonadotropin exposure may lead to nuclear and cytoplasmic immaturity. Yang *et al*. (2019a) conducted a retrospective cohort study and found that oocyte maturation rates were significantly lower with shorter stimulation periods (<6 days) compared to longer stimulation periods (> 7 days) in normal responders. It is still unclear whether immature oocytes are generated from small antral follicles at the time of oocyte retrieval or large preovulatory follicles that do not respond to hCG (Lee *et al*., 2012). Clinicians often struggle to increase the length of stimulation and recover more mature oocytes or reduce the time to pregnancy by avoiding freezing all embryos because of a premature progesterone rise. Some authors have performed a randomized controlled trial designed to evaluate the number of mature oocytes at retrieval. They found that patients with a high (>1.5ng/ml) elevation in serum progesterone and a 24-hour delay in oocyte maturation trigger were associated with a significantly higher number of mature oocytes, fertilized oocytes, and good quality embryos. However, this approach did not yield a higher number of mature oocytes in patients with moderately elevated serum progesterone levels (>1ng/ml and <1.5ng/ml) (Vandekerckhove *et al*., 2014). Authors such as Davar *et al*. (2017) did not find differences in the number of mature oocytes from delaying the hCG trigger.

Presently, mild ovarian stimulation protocols seem to be part of the measures that might palliate these effects. The idea that oral agents might prevent excessive responses along with patient-friendly ovarian stimulation is promising. Nevertheless, some authors have reached different conclusions. Yang *et al*. (2019b) conducted a randomized parallel controlled study and found that letrozole supplementation did not reduce the rising progesterone levels in the late follicular phase in high responders. Labarta *et al*. (2018) found the same results in women with poor ovarian response (according to the Bologna criteria), in that no significant differences were found between mild and conventional ovarian stimulation in terms of progesterone levels on hCG day.

## IMPACT OF PREMATURE PROGESTERONE RISE AND ENDOMETRIAL RECEPTIVITY

Endometrial receptivity is a tightly regulated process, with estrogen and progesterone as its major regulators. Estrogen commands the proliferative phase of the menstrual cycle, while the secretory phase is mainly under the control of progesterone. Embryo implantation implies the apposition, attachment, and adhesion of the blastocyst to the luminal epithelium (LE) (Kelleher *et al*., 2018). Several endometrial receptivity markers have been described in association with this event. The most studied markers are transcriptional factors such as HOXA genes. HOXA-10 and HOXA-11, observed in the mid-luteal phase of the human menstrual cycle, have equally important roles in implantation. In conjunction with HOXA genes, there is a family of proteins with almost 21 isoforms that possess diverse functions, including redox reactions, protein folding, and chaperone activity. Aside from these functions, they have been described to play an essential rle in the implantation process by modulating embryo attachment (Fernando *et al*., 2021).

Several previous studies (Al-Azemi *et al*., 2012; Bosch *et al*., 2010; Venetis *et al*., 2013; Labarta *et al*., 2011) demonstrated the harmful effects of high progesterone levels on endometrial receptivity, an event that explains, at least partially, adverse reproductive outcomes in women undergoing IVF cycles. Progesterone is crucial for endometrial glandular development and initiates the modifications needed for implantation. In a prospective study, endometrial biopsies from women with premature progesterone rise revealed a significantly advanced histological staging, by 0.3 to 1.0 days. Other authors reached similar conclusions respective to histological dating (Van Vaerenbergh *et al*., 2011). The proportion of glandular- stroma asynchrony was also significantly higher. Liu *et al*. (2015) found that high progesterone levels on hCG day were associated with the number of uterine natural killer (uNK) cells. Considering that uNK cells do not express progesterone receptors, it is reasonable to believe that progesterone might exert its effects indirectly via cytokines or other soluble factors produced by uterine stromal cells (Agostinis *et al*., 2019). Carlino *et al*. (2012) demonstrated that uterine stromal cells from fertile or menopausal women could release chemerin in response to progesterone and 17-β estradiol, therefore supporting the migration of peripheral NK cells through stromal cells. In an animal model, the cytokine leukemia inhibitory factor (LIF) is strongly expressed in the endometrium during the implantation window in response to the nidatory surge in estrogen from the ovary (Kelleher *et al*., 2018). LIF is primarily expressed in the glandular epithelium, and it is required for implantation since it initiates embryo attachment (Kelleher *et al*., 2018). In progesterone-treated pregnant mice, the level of LIF mRNA expression was inhibited compared to controls. Furthermore, progesterone-treated pregnant mice had a significantly lower number of implantation sites compared to controls (Liang *et al*., 2018). Higher progesterone levels on hCG day led to significantly higher uNK cell counts in the endometrium (7 days after hCG administration) than normal progesterone concentrations (Liu *et al*., 2015). However, the role of uNK cells in the endometrium is still unclear, and further research is needed to establish their effects on the implantation process. Conjointly excessive progesterone levels affect the expression of estrogen (ER) and progesterone receptors (PR). In a mouse model, Liang *et al*. (2018) demonstrated that excessive progesterone levels reduced the expression of progesterone and estrogen receptors, and were associated with significantly decreased expression of well-known markers of human *in vitro* decidualization in a dosage-dependent manner. These results supported the findings published by Van Vaerenbergh *et al*. (2011), which showed significant impacts on endometrial gene expression on a crucial protease for insulin-like growth-factor-binging protein 4, known as pregnancy-associated plasma protein-A (PAPP-A). The results published by Van Vaerenbergh *et al*. (2011) provide evidence that there is a small number of divergent endometrial gene expression between <0.9ng/ml to 1.5ng/ml and a sizable amount of differentially expressed genes in patients with more than 1.5ng/ml.

Therefore, this evidence supports the harmful effect of high progesterone levels on the implantation process in a dose-dependent fashion. Evidence is accumulating on the different effects on ovarian response produced by different progesterone level ranges. In a retrospective cohort study, Oktem *et al*. (2019) found that high responders were not free from the detrimental effects of premature progesterone elevation; the difference was that deleterious effects in high responders started at a higher threshold. Oktem *et al*. (2019) observed that in poor responders, a significant reduction in the probability of pregnancy appeared in the 1.5 to 1.9ng/ml range, while in normal responders a harmful effect began at 3.0 to 3.4ng/ml. High responders suffered from deleterious effects when progesterone reached 4.0 to 4.4ng/ml.

## PREMATURE PROGESTERONE RISE AND EMBRYO QUALITY

In an apparent contradiction with previous findings, published data has pointed out that there is an association between the progesterone level on hCG day and the rate of top embryo quality (TEQ). It seems that patients with elevated progesterone levels during the late follicular phase are at risk of not having top quality blastocysts. According to Huang *et al*. (2016), the top quality embryo rate was significantly different in individuals with serum progesterone levels <2.0ng/ml before oocyte maturation. Some authors reached different conclusions and found that premature progesterone rise affects oocyte quality and therefore embryo quality according to ovarian response. Poor ovarian responders are the most affected by premature progesterone rise, with a significant reduction in high quality embryo rates compared to poor responders without elevated progesterone. Intermediate and high ovarian responders were not significantly different in terms of high quality embryo rate between individuals with and without elevated progesterone levels (Bu *et al*., 2014). A few authors concluded that elevated progesterone levels affect live birth rates regardless of protective features such as age and good ovarian response (Hill *et al*., 2015).

In animal models such as cattle and horses, the follicles remain sensitive to gonadotropins even when they are growing under high levels of progesterone and/or long lasting exposure to progesterone. Animal reproduction studies showed that progesterone-treated sheep had greater numbers of harvested oocytes and embryos, higher fertilization rates, and higher proportions of grade-1 embryos (Cuadro *et al*., 2018). Other authors found no significant differences in the number of MII oocytes retrieved, fertilization, blastocyst or euploid blastocyst rates between follicular and luteal phase stimulation (Ubaldi *et al*., 2016; Kuang *et al*., 2014; Zhang *et al*., 2017). Zhang *et al*. (2017) found that embryos produced during the luteal phase resulted in higher implantation rates than embryos obtained with follicular phase stimulation.

Evidence on double stimulation protocols is growing. Vaiarelli *et al*. (2018) found that high progesterone environments might yield competent oocytes, collected from follicular and luteal phase stimulations, which ultimately reach similar outcomes in terms of fertilization, blastulation, and euploidy rates, as well as good clinical outcomes after single blastocyst transfers. Double stimulation provides more euploid blastocysts in 65.5% of the patients against 42% of the patients if only follicular phase stimulation is performed (Vaiarelli *et al*., 2020).

## References

[r1] Agostinis C, Mangogna A, Bossi F, Ricci G, Kishore U, Bulla R (2019). Uterine Immunity and Microbiota: A Shifting Paradigm. Front Immunol.

[r2] Allegra A, Marino A, Volpes A, Coffaro F, Scaglione P, Gullo S, La Marca A (2017). A randomized controlled trial investigating the use of a predictive nomogram for the selection of the FSH starting dose in IVF/ICSI cycles. Reprod Biomed Online.

[r3] Alviggi C, Conforti A, Esteves SC, Andersen CY, Bosch E, Bühler K, Ferraretti AP, De Placido G, Mollo A, Fischer R, Humaidan P, International Collaborative Group for the Study of r-hLH (iCOS-LH) (2018). Recombinant luteinizing hormone supplementation in assisted reproductive technology: a systematic review. Fertil Steril.

[r4] Al-Azemi M, Kyrou D, Kolibianakis EM, Humaidan P, Van Vaerenbergh I, Devroey P, Fatemi HM (2012). Elevated progesterone during ovarian stimulation for IVF. Reprod Biomed Online.

[r5] Arvis P, Lehert P, Guivarc'h-Levêque A (2019). Both high and low HCG day progesterone concentrations negatively affect live birth rates in IVF/ICSI cycles. Reprod Biomed Online.

[r6] Bianchi S, Macchiarelli G, Micara G, Linari A, Boninsegna C, Aragona C, Rossi G, Cecconi S, Nottola SA (2015). Ultrastructural markers of quality are impaired in human metaphase II aged oocytes: a comparison between reproductive and in vitro aging. J Assist Reprod Genet.

[r7] Bosch E, Labarta E, Crespo J, Simón C, Remohí J, Jenkins J, Pellicer A (2010). Circulating progesterone levels and ongoing pregnancy rates in controlled ovarian stimulation cycles for in vitro fertilization: analysis of over 4000 cycles. Hum Reprod.

[r8] Bu Z, Zhao F, Wang K, Guo Y, Su Y, Zhai J, Sun Y (2014). Serum progesterone elevation adversely affects cumulative live birth rate in different ovarian responders during in vitro fertilization and embryo transfer: a large retrospective study. PLoS One.

[r9] Carlino C, Trotta E, Stabile H, Morrone S, Bulla R, Soriani A, Iannitto ML, Agostinis C, Mocci C, Minozzi M, Aragona C, Perniola G, Tedesco F, Sozzani S, Santoni A, Gismondi A (2012). Chemerin regulates NK cell accumulation and endothelial cell morphogenesis in the decidua during early pregnancy. J Clin Endocrinol Metab.

[r10] Chalumeau C, Moreau J, Gatimel N, Cohade C, Lesourd F, Parinaud J, Leandri R (2018). Establishment and validation of a score to predict ovarian response to stimulation in IVF. Reprod Biomed Online.

[r11] Cuadro F, Dos Santos-Neto PC, Pinczak A, Barrera N, Crispo M, Menchaca A (2018). Serum progesterone concentrations during FSH superstimulation of the first follicular wave affect embryo production in sheep. Anim Reprod Sci.

[r12] Davar R, Naghshineh E, Neghab N (2017). The effect of 24 hours delay in oocyte maturation triggering in IVF/ICSI cycles with antagonist protocol and not-elevated progesterone: A randomized control trial. Int J Reprod Biomed.

[r13] de Ziegler D, Fraisse T, de Candolle G, Vulliemoz N, Bellavia M, Colamaria S (2007). Outlook: Roles of FSH and LH during the follicular phase: insight into natural cycle IVF. Reprod Biomed Online.

[r14] de Ziegler D, Andersen CY, Stanczyk FZ, Ayoubi JM (2018). Endocrine mechanisms and assay issues in premature progesterone elevation in assisted reproductive technology. Fertil Steril.

[r15] Drakopoulos P, Racca A, Errázuriz J, De Vos M, Tournaye H, Blockeel C, Pluchino N, Santos-Ribeiro S (2019). The role of progesterone elevation in IVF. Reprod Biol.

[r16] Dumesic DA, Meldrum DR, Katz-Jaffe MG, Krisher RL, Schoolcraft WB (2015). Oocyte environment: follicular fluid and cumulus cells are critical for oocyte health. Fertil Steril.

[r17] Fernando SR, Kottawatta KSA, Jiang L, Chen X, Cheng KW, Wong BP, Ng EH, Yeung WS, Lee KF (2021). Differential expression of protein disulfide isomerase (PDI) in regulating endometrial receptivity in humans. Reprod Biol.

[r18] Griffin D, Feinn R, Engmann L, Nulsen J, Budinetz T, Benadiva C (2014). Dual trigger with gonadotropin-releasing hormone agonist and standard dose human chorionic gonadotropin to improve oocyte maturity rates. Fertil Steril.

[r19] Harada T, Katagiri C, Takao N, Toda T, Mio Y, Terakawa N (1996). Altering the timing of human chorionic gonadotropin injection according to serum progesterone (P) concentrations improves embryo quality in cycles with subtle P rise. Fertil Steril.

[r20] Hill MJ, 4th Royster GD, Healy MW, Richter KS, Levy G, DeCherney AH, Levens ED, Suthar G, Widra E, Levy MJ (2015). Are good patient and embryo characteristics protective against the negative effect of elevated progesterone level on the day of oocyte maturation?. Fertil Steril.

[r21] Huang B, Ren X, Wu L, Zhu L, Xu B, Li Y, Ai J, Jin L (2016). Elevated Progesterone Levels on the Day of Oocyte Maturation May Affect Top Quality Embryo IVF Cycles. PLoS One.

[r22] Hugues JN (2012). Impact of 'LH activity' supplementation on serum progesterone levels during controlled ovarian stimulation: a systematic review. Hum Reprod.

[r23] Kelleher AM, Milano-Foster J, Behura SK, Spencer TE (2018). Uterine glands coordinate on-time embryo implantation and impact endometrial decidualization for pregnancy success. Nat Commun.

[r24] Kuang Y, Chen Q, Hong Q, Lyu Q, Ai A, Fu Y, Shoham Z (2014). Double stimulations during the follicular and luteal phases of poor responders in IVF/ICSI programmes (Shanghai protocol). Reprod Biomed Online.

[r25] Labarta E, Martínez-Conejero JA, Alamá P, Horcajadas JA, Pellicer A, Simón C, Bosch E (2011). Endometrial receptivity is affected in women with high circulating progesterone levels at the end of the follicular phase: a functional genomics analysis. Hum Reprod.

[r26] Labarta E, Marin D, Remohí J, Bosch E (2018). Conventional versus minimal ovarian stimulation: an intra-patient comparison of ovarian response in poor-responder women according to Bologna Criteria. Reprod Biomed Online.

[r27] Lawrenz B, Beligotti F, Engelmann N, Gates D, Fatemi HM (2016). Impact of gonadotropin type on progesterone elevation during ovarian stimulation in GnRH antagonist cycles. Hum Reprod.

[r28] Lawrenz B, Fatemi HM (2017). Effect of progesterone elevation in follicular phase of IVF-cycles on the endometrial receptivity. Reprod Biomed Online.

[r29] Lawrenz B, Melado L, Fatemi H (2018a). Premature progesterone rise in ART-cycles. Reprod Biol.

[r30] Lawrenz B, Labarta E, Fatemi H, Bosch E (2018b). Premature progesterone elevation: targets and rescue strategies. Fertil Steril.

[r31] Lawrenz B, Long J, Stoop D, Missou I, Fatemi H (2018c). Impact of stimulation duration and gonadotropin type on the incidence of premature progesterone elevation - a retrospective analysis of the Ensure data. Gynecol Endocrinol.

[r32] Lee HJ, Jee BC, Suh CS, Kim SH, Moon SY (2012). Oocyte maturity in relation to woman's age in in vitro fertilization cycles stimulated by single regimen. Yonsei Med J.

[r33] Liang YX, Liu L, Jin ZY, Liang XH, Fu YS, Gu XW, Yang ZM (2018). The high concentration of progesterone is harmful for endometrial receptivity and decidualization. Sci Rep.

[r34] Liu L, Sailan S, Li T, Mariee N, Laird S, Jiang Z, Zhang S (2015). The effect of a high progesterone concentration before oocyte retrieval on the peri-implantation endometrium. Reprod Biomed Online.

[r35] Luborsky JL, Thiruppathi P, Rivnay B, Roussev R, Coulam C, Radwanska E (2002). Evidence for different aetiologies of low estradiol response to FSH: age-related accelerated luteinization of follicles or presence of ovarian autoantibodies. Hum Reprod.

[r36] Nisal A, Diwekar U, Bhalerao V (2020). Personalized medicine for in vitro fertilization procedure using modeling and optimal control. J Theor Biol.

[r37] Oktem O, Yakin K, Oguz SY, Isiklar A, Balaban B, Urman B (2019). High responders are not exempt from detrimental effects of prematurely rising progesterone levels in fresh embryo transfer cycles. Reprod Biomed Online.

[r38] Papaleo E, Zaffagnini S, Munaretto M, Vanni VS, Rebonato G, Grisendi V, Di Paola R, La Marca A (2016). Clinical application of a nomogram based on age, serum FSH and AMH to select the FSH starting dose in IVF/ICSI cycles: a retrospective two-centres study. Eur J Obstet Gynecol Reprod Biol.

[r39] Schneyer AL, Fujiwara T, Fox J, Welt CK, Adams J, Messerlian GM, Taylor AE (2000). Dynamic changes in the intrafollicular inhibin/activin/follistatin axis during human follicular development: relationship to circulating hormone concentrations. J Clin Endocrinol Metab.

[r40] Stanczyk FZ, Archer DF, Bhavnani BR (2013). Ethinyl estradiol and 17β-estradiol in combined oral contraceptives: pharmacokinetics, pharmacodynamics and risk assessment. Contraception.

[r41] Ubaldi FM, Capalbo A, Vaiarelli A, Cimadomo D, Colamaria S, Alviggi C, Trabucco E, Venturella R, Vajta G, Rienzi L (2016). Follicular versus luteal phase ovarian stimulation during the same menstrual cycle (DuoStim) in a reduced ovarian reserve population results in a similar euploid blastocyst formation rate: new insight in ovarian reserve exploitation. Fertil Steril.

[r42] Vaiarelli A, Cimadomo D, Trabucco E, Vallefuoco R, Buffo L, Dusi L, Fiorini F, Barnocchi N, Bulletti FM, Rienzi L, Ubaldi FM (2018). Double Stimulation in the Same Ovarian Cycle (DuoStim) to Maximize the Number of Oocytes Retrieved From Poor Prognosis Patients: A Multicenter Experience and SWOT Analysis. Front Endocrinol (Lausanne).

[r43] Vaiarelli A, Cimadomo D, Petriglia C, Conforti A, Alviggi C, Ubaldi N, Ledda S, Ferrero S, Rienzi L, Ubaldi FM (2020). DuoStim - a reproducible strategy to obtain more oocytes and competent embryos in a short time-frame aimed at fertility preservation and IVF purposes. A systematic review. Ups J Med Sci.

[r44] van Tilborg TC, Oudshoorn SC, Eijkemans MJC, Mochtar MH, van Golde RJT, Hoek A, Kuchenbecker WKH, Fleischer K, de Bruin JP, Groen H, van Wely M, Lambalk CB, Laven JSE, Mol BWJ, Broekmans FJM, Torrance HL, OPTIMIST study group (2017). Individualized FSH dosing based on ovarian reserve testing in women starting IVF/ICSI: a multicentre trial and cost-effectiveness analysis. Hum Reprod.

[r45] Van Vaerenbergh I, Fatemi HM, Blockeel C, Van Lommel L, In't Veld P, Schuit F, Kolibianakis EM, Devroey P, Bourgain C (2011). Progesterone rise on HCG day in GnRH antagonist/rFSH stimulated cycles affects endometrial gene expression. Reprod Biomed Online.

[r46] Vandekerckhove F, Gerris J, Vansteelandt S, De Baerdemaeker A, Tilleman K, De Sutter P (2014). Delaying the oocyte maturation trigger by one day leads to a higher metaphase II oocyte yield in IVF/ICSI: a randomised controlled trial. Reprod Biol Endocrinol.

[r47] Vázquez AC, Rodríguez JMAG, Algara ALC, García JDM (2021). Correlation between biochemical, ultrasonographic and demographic parameters with ovarian response to IVF/ICSI treatments in Mexican women. JBRA Assist Reprod.

[r48] Venetis CA, Kolibianakis EM, Bosdou JK, Tarlatzis BC (2013). Progesterone elevation and probability of pregnancy after IVF: a systematic review and meta-analysis of over 60 000 cycles. Hum Reprod Update.

[r49] Wu YG, Barad DH, Kushnir VA, Lazzaroni E, Wang Q, Albertini DF, Gleicher N (2015). Aging-related premature luteinization of granulosa cells is avoided by early oocyte retrieval. J Endocrinol.

[r50] Yang YC, Li YP, Pan SP, Chao KH, Chang CH, Yang JH, Chen SU (2019a). The different impact of stimulation duration on oocyte maturation and pregnancy outcome in fresh cycles with GnRH antagonist protocol in poor responders and normal responders. Taiwan J Obstet Gynecol.

[r51] Yang X, Lin G, Lu G, Gong F (2019b). Letrozole supplementation during controlled ovarian stimulation in expected high responders: a pilot randomized controlled study. Reprod Biol Endocrinol.

[r52] Yding Andersen C (2002). Effect of FSH and its different isoforms on maturation of oocytes from pre-ovulatory follicles. Reprod Biomed Online.

[r53] Younis JS (2019). The role of progesterone/estradiol ratio in exploring the mechanism of late follicular progesterone elevation in low ovarian reserve women. Med Hypotheses.

[r54] Zhang Q, Guo XM, Li Y (2017). Implantation rates subsequent to the transfer of embryos produced at different phases during double stimulation of poor ovarian responders. Reprod Fertil Dev.

